# EBUS-TBNA在肺部疾病（包括肺癌分期）中的作用综述

**DOI:** 10.3779/j.issn.1009-3419.2010.05.07

**Published:** 2010-05-20

**Authors:** David FIELDING, Farzad BASHIRZADEH, Phan NGUYEN, Alan HODGSON, Daniel JAMES, 齐 吴, 楠 吴

**Affiliations:** 1 Department of Thoracic Medicine, Royal Brisbane and Womens' Hospital, Herston, Australia; 2 Department of Anatomical Pathology, Pathology Queensland, Royal Brisbane and Womens' Hospital, Herston, Australia; 3 北京肿瘤医院内镜科; 4 天津医科大学总医院，天津市肺癌研究所，天津市肺癌转移与肿瘤微环境重点实验室

**Keywords:** 支气管镜, 支气管内超声, 肺癌, 结节病和其它肉芽肿, 分期

## Abstract

本综述重点阐述EBUS-TBNA在日常肺部疾病诊疗中的作用。所列举的病例为EBUS-TBNA的常见适应征，包括：①肺癌分期；②确诊胸部恶性淋巴结；③诊断中央型肺部肿块；④诊断肺结节病；⑤诊断炎性/良性胸部淋巴结。这项技术应用广泛，有经验的支气管镜医师经过适当的训练后，该技术将成为支气管镜检查的一部分。

## 前言

目前，经支气管镜引导针吸活检（endobronchial ultrasound-guided transbronchial needle aspiration, EBUS-TBNA）是胸科医生认可的诊断方法^[1, 2]^。最初，支气管镜常用于诊断支气管内病变，现在，我们可借助一些手段将视野延伸至支气管外。通过纤维支气管镜使用可弯曲细针的盲法TBNA始于20世纪80年代早期^[3, 4]^。尽管许多研究表明其安全有效，但其仅为部分胸科医生接受，且大量调查显示仅10%-30%的胸科医生采用了该项技术^[5]^。即使在应用该技术的30余年间未见严重出血报道，但低成功率和担心穿透血管仍是其未得到广泛应用的主要原因。EBUS-TBNA直接针对这些难点。在大多数方面，与标准的TBNA相比，EBUS方法有助于更好地穿透支气管壁（由于存在活检管道，TBNA穿刺针形成向前的成角），可以显示淋巴结内穿刺针的确切位置，并可见周围血管，特别是肺门和低位气管旁区域的血管。其学习曲线相对较短，在20例-30例（或更少）操作后可获得持续良好的细胞学诊断率^[6]^。

已经发表的关于EBUS-TBNA的大量优秀综述涵盖了方法、结果和适应征^[7, 8]^。本综述将呈现代表其最常见适应征的5个病例并讨论相关文献。其中4例出现于一个月内。在本院，每月行20例-25例EBUS-TBNA，已有近600例操作经验。

## EBUS-TBNA的解剖学和方法学

最新的国际肺癌研究协会（IASLC）肺癌分期指南更好地阐明了淋巴结位置分区，这是一个重要的起点^[9]^。熟悉一些与支气管相关的关键结构的位置十分必要。右侧包括：上腔静脉（SVC）、奇静脉、右肺动脉和右下叶肺动脉。左侧包括：升主动脉、左肺动脉、左下叶肺动脉和食管。学习这些的有效方法是上下滚动数字化CT片，查看上述结构在何处与支气管接触（如图 1a，图 1b）。更有效的是水平方向旋转CT片，这种影像相当于站在患者后方进行支气管镜检查。Wang的支气管镜下淋巴结位点表面标记^[3, 4]^和Ko关于CT下淋巴结定位的文章也十分有用^[10]^。针对每一个站点的“解剖方位识别技术”是这些方法中最好的^[7]^。我们建议从隆突下穿刺开始操作，随经验的积累扩展至其它位置。

**1:**
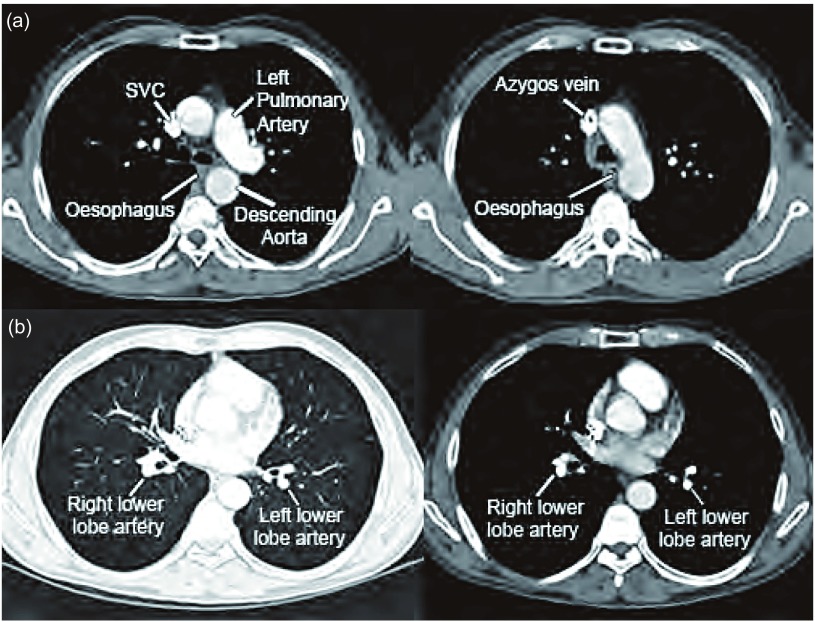
TBNA相关主要解剖结构的隆突（a）和肺门（b）CT图像。SVC：上腔静脉。 Main relevant anatomical structures for transbronchial needle aspiration on computerized tomography at the (a) carina and (b) lower hilar regions. SVC, Superior vena cava.

大量优秀的出版物描述了EBUS-TBNA方法的要点^[11-14]^。操作可在局部麻醉联合持续镇静或全身麻醉面罩吸氧下成功进行。初学者往往采用后一种麻醉方法。简而言之，专用的EBUS-TBNA支气管镜须配套使用专用的EBUS-TBNA穿刺针。准备好穿刺针及超声探头顶端安装的小气囊后，常规进镜，经支气管树将内镜放置于穿刺结节邻近点。气囊内充入少量生理盐水以改善超声图像质量。在将穿刺针插入活检通道过程中，当发现淋巴结并观察到其与邻近血管分离时，助手持镜并将其固定在适当位置，操作者在直接超声引导下轻柔进针至淋巴结内。空心针用于将针顶端杂物拨离并吸入组织。通过多次进出淋巴结不同区域获得样本。取出穿刺针后将样本置于玻片或生理盐水中处理。通常现场有一位细胞学技师评价样本质量以及用以诊断的组织量是否足够。

## 病例报道及文献综述

例1  肺癌分期

78岁，男性，支气管灌洗细胞学诊断为左下肺4 cm腺癌，胸部CT显示隆突下（7L）、左侧气管旁（4L）、右侧气管旁（4 R）区域淋巴结肿大（直径1 0 mm - 1 3 mm），PET扫查上述区域淋巴结阳性（图 2a）。通过EBUS-TBNA对4R区域淋巴结进行取样。现场细胞学诊断第一次穿刺结果阳性（图 2b，图 2c）。分期为N3，共用时20 min。

**2:**
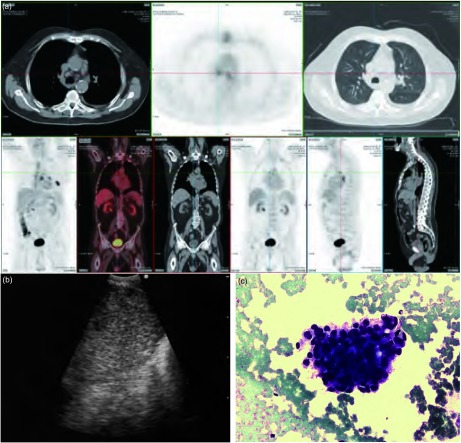
例1。（a）：PET图像；（b）：4R区淋巴结的支气管内超声图像；（c）：支气管针吸活检组织的细胞学涂片，10倍视野，在红细胞中可见一簇恶性细胞。 Case 1. (a) Positron emission tomography scan. (b) Endobronchial ultrasound appearance of 4R lymph node. (c) Cytology smear of transbronchial needle aspiration material showing a clump of malignant cells among erythrocytes (Pap), 10x. A.

肺癌分期是EBUS-TBNA最常见适应征之一^[15]^。其能够到达肺门和叶间区域，而纵隔镜仅能到达气管旁，偶尔到达隆突下区域^[7]^。对于淋巴结而言，PET和CT诊断均存在假阳性和假阴性结果。如果淋巴结的准确诊断可改变治疗策略，尤其是根据一个单发肿大淋巴结可决定是否应该行手术治疗时，需要获取淋巴结组织。

Yasufuku的文章是EBUS-TBNA用于肺癌分期发展上的里程碑^[16]^。102例可切除肺癌患者在开胸外科手术分期前行CT、PET和EBUS-TBNA检查。三种方法的相对敏感性和特异性分别为77%、80%和92%。诊断准确性分别为61%、73%和98%。EBUS-TBNA除可到达更广泛的淋巴结区域外，还可以从直径5 mm的淋巴结中取样。一项涉及1 299例患者的EBUS-TBNA用于肺癌分期的*meta*分析显示其总的敏感性为93%，特异性为100%^[17]^。Rintoul等发表了PET阳性淋巴结的EBUS-TBNA检测结果^[18]^。在曾接受EBUS-TBNA和外科分期的109例患者中，77例患者的恶性淋巴结得到确诊。7例EBUS-TBNA阴性而外科活检恶性（TBNA假阴性），其中4例取样错误，3例检测错误。因此，他们建议PET阳性而EBUS-TBNA阴性的淋巴结应行外科活检（大多数与根治性切除术同时进行）。

Herth等提供了影像学及PET正常纵隔的EBUS-TBNA分期资料^[19]^。对97例可切除肺癌患者的156枚淋巴结（5 mm-10 mm）取样，9例检出恶性，1例漏诊（经切除淋巴结组织学证实）。尽管如此，但目前大多数医学中心认为可切除肺癌患者CT显示无纵隔淋巴结肿大，并且PET扫描阴性，无需在外科切除前优先行EBUS-TBNA分期。

2005年，Rintoul等和Vilmann等报告EBUS可联合EUS用于纵隔分期^[20, 21]^。在Vilmann的研究中，33例患者行EUS和EBUS分期，EUS取样59枚淋巴结，EBUS取样60枚，EUS发现26例恶性，EBUS发现28例恶性。EBUS比EUS-FNA（1区、气管前、2R、4L、4R、7区和10L）多发现11枚阳性淋巴结。EUS（2R、4R、4L、7区和左肾上腺）比EBUS多发现12枚恶性淋巴结。提示EBUS是获取气管前、10区和11区淋巴结的唯一方式；反之，EUS是获取8区（2例）、9区（1例）和肾上腺区域淋巴结的唯一方式。据此，他们推断两种方法具有叠加效应。尽管全球许多医学中心具备联合使用两种检查方法的条件，但如何将其转化为临床实践尚不清楚，关键问题在于，对于患者个体而言，检测的个体淋巴结数目的增加是否会改变分期？许多患者最终必须明确这一问题。

在分期过程中，穿刺取样一般从N3区域淋巴结到远端N2、近端N2、再到N1区域淋巴结。最好行现场细胞学检查，一旦细胞学检查呈阳性即可停止操作。如果在分期的临床研究中不能进行现场细胞学检查，因生理盐水冲洗针腔无法清除上一部位穿刺造成的污染，故穿刺每一区域均应更换穿刺针。在日常检查中，人们公认其检查费用昂贵。但只要严格按照顺序取样，每一位患者的分期诊断均不会被升级。

例2  淋巴结活检诊断肺癌

76岁，老年男性，伴右侧胸痛症状。既往石棉接触史和35包年的吸烟史；5年前因肾癌行肾切除术。CT显示右下叶胸膜处可疑肿块，伴胸腔积液（图 2a）。此外，右下肺门（R11i）和隆突下（7R）淋巴结肿大。既往支气管镜检查管腔内未见占位，支气管灌洗结果阴性。PET扫查显示肿块低摄取，但右肺门及隆突下淋巴结高摄取（图 3a）。我们对右下肺门（R11i）区域淋巴结行EBUS-TBNA并取样，第一针穿刺组织的现场细胞学检查即符合非小细胞肺癌，随后免疫组化染色TTF1呈阳性。因原发病灶无法切除，未行隆突下淋巴结取样，整个过程用时15 min。

**3:**
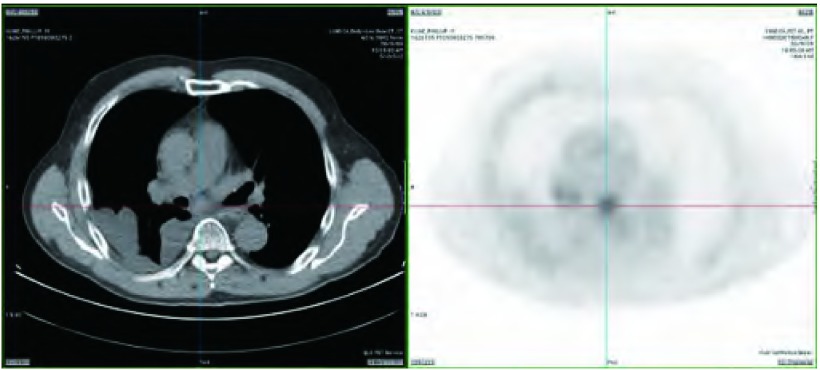
例2。PET图像。 Case 2. Positron emission tomography scan.

一般而言，在诊断纵隔淋巴结肿大时，大量前瞻性研究显示EBUS-TBNA具有高敏感性（85%-96%）、高特异性（100%）以及高诊断准确性（89%-97%）^[1]^。本病例中，高度可疑的N1和N2期淋巴结位于易于到达的区域，因此对其取样，而不对更难以到达的周围型肿块取样。如果是肾癌转移病灶，TBLB后可能发生出血。同时，如果其为间皮瘤，TBLB可能无法到达。我们发表了几篇关于疑似周围型肺癌患者首先行EBUS-TBNA的文章^[22]^。因周围型肿块未被取样，其可能与淋巴结细胞的来源不同，这一观点尚存争议。但我们认为两处阴影（肿块和淋巴结）不太可能仅为影像学异常，尤其当PET显示身体其它部位无明显摄取时。这一方法并且可以免除CT FNA等其它操作。

例3  原发肺癌肿块的活检

40岁，女性，出现胸背中上部疼痛，既往吸烟史40包年。CT显示左上叶高位肿物，与气管左后外侧紧邻（图 4a）。无区域淋巴结肿大。支气管镜显示声带下3 cm气管外压性改变，质软，无气管腔内病变，行EBUSTBNA于声带下3 cm气管7点处进针穿刺（图 4b）。现场细胞学检查第一针穿刺组织确诊为非小细胞肺癌。整个过程用时10 min。

**4:**
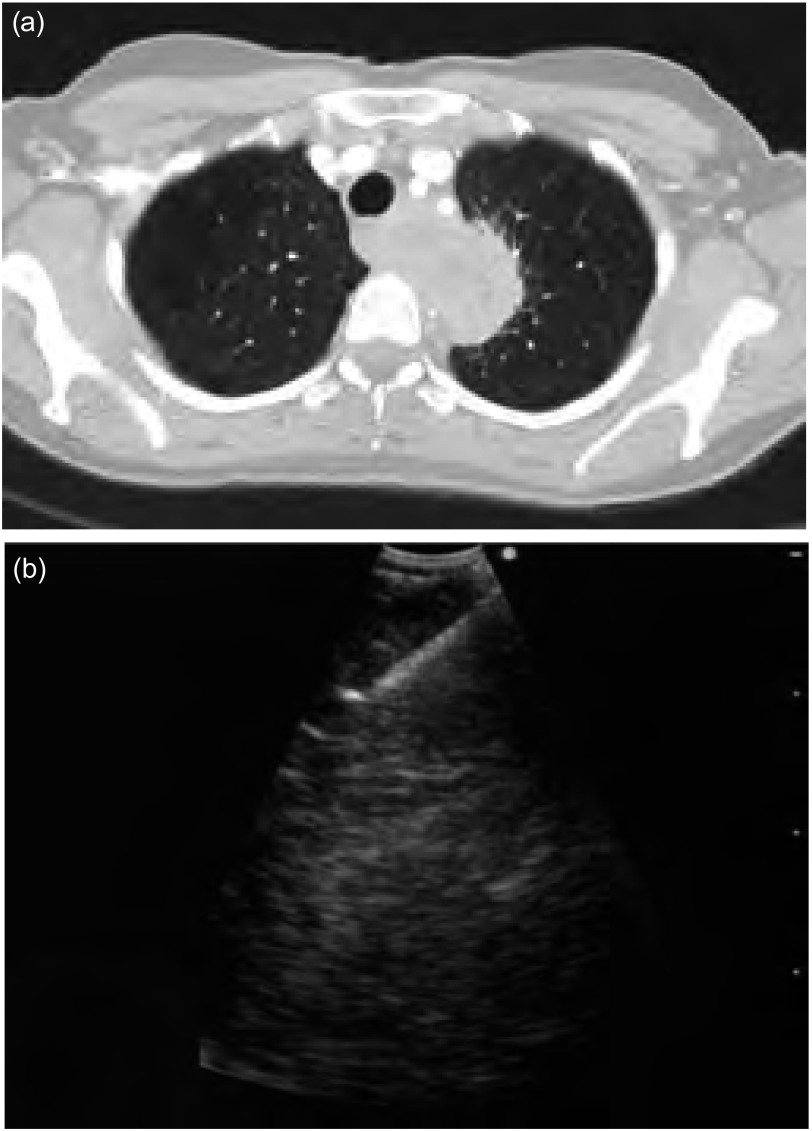
例3。（a）：胸部CT图像；（b）：超声图像，肿块占据图像大部，可见针吸活检通道。 Case 3. (a) Chest computerized tomography. (b) EBUS appearance showing the large mass occupying most of the ultrasound view, with EBUS-TBNA needle inserted for sampling.

许多学者指出支气管腔内无病灶时，采用EBUSTBNA诊断原发肺肿块具有优势^[23, 24]^。肿块正对着大气道时，可避免行CT FNA和TBLB或外科前纵隔切开等其它更难的操作。当肿块邻近段支气管，如EBUS-TBNA探头可以到达肿块，其较之TBLB更容易。显然，这一方法的限制因素为相应支气管的管径。EBUS-TBNA探头最大外径为6.9 mm。一般认为如果CT扫查显示通向肿块的支气管口径约4 mm，探头即可通过并行EBUS-TBNA。因双肺上叶亚段开口锐角较多，该探头无法通过而导致穿刺活检困难。

例4  结节病

48岁，男性，可见持续性淋巴结肿大，合并轻度持续性胸壁疼痛，在无病理诊断情况下已开始3个月的针对结节病的激素治疗，胸部CT显示双侧肺门和纵隔淋巴结肿大，肺实质无病变。激素治疗后CT无变化，行隆突下及右下肺门（11i）淋巴结EBUS-TBNA取样（图 5a）。EBUS显示淋巴结内大量整齐的隔膜，多普勒显示内部有小血管。抽吸活检组织现场组织细胞学显示无干酪性肉芽肿，感染性有机体染色阴性。另1例结节病病理如图（图 5b，图 5C）所示，气管内活检阴性。对此解释为，该患者确实罹患结节病，持续治疗且症状缓解。

**5:**
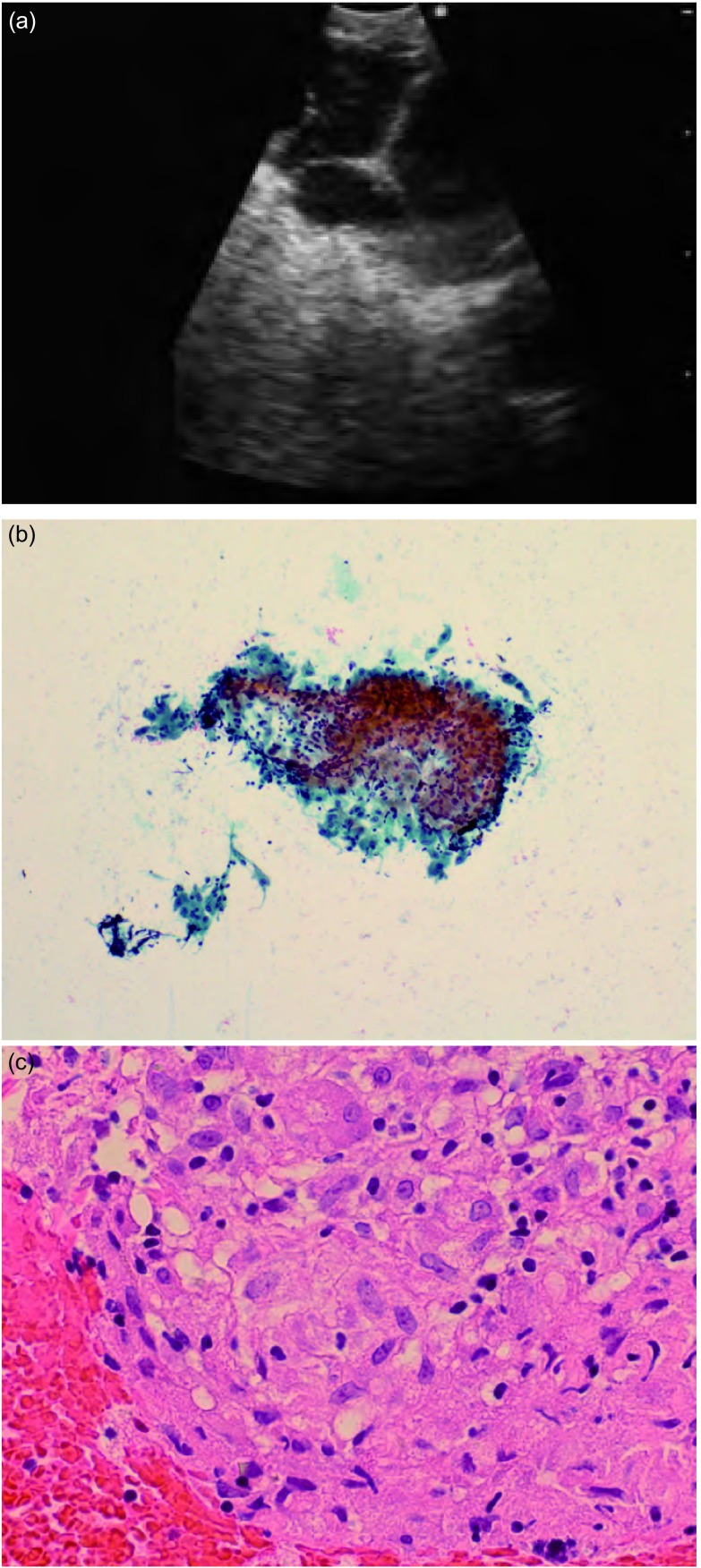
例4。（a）：肺结节病淋巴结特异性超声图像；（b）：穿刺组织细胞学涂片，10倍视野，显示一个肉芽肿；（c）：穿刺组织（HE染色，40倍视野）。 Case 4. (a) EBUS appearance of sarcoid lymph node showing typical septations. (b) Cytology smear of TBNA material(Pap), 10x. showing a granuloma. (c) Histology of TBNA material (H & E, 40x) detail of granuloma showing histiocytes.

此病例之后，我们常规要求对疑似结节病例行现场病理检查，如果吸取物显示肉芽肿则停止操作。此外，我们常将活检组织置于浸泡于福尔马林中的小方形滤纸上以便行组织学切片。22 G TBNA穿刺针可获得阳性结果，现在更粗的（21 G）穿刺针的出现将提高诊断率。

肉芽肿为上皮样巨噬细胞聚集成团^[25]^，细胞核呈圆形或椭圆形，可见一些足迹样或鞋印样延伸，即使是多形性细胞核，其核膜光滑，含有细粒状染色质，胞浆淡色、丰富，细胞边界不清。与结核性肉芽肿不同，肺结节病玻片干净，仅残留血迹，无坏死碎屑出现^[26]^。

很多医师描述结节病淋巴结EBUS特征性表现为：结节分隔或平直的血管，这种现象支持结节病诊断。（Kurimoto N, World Congress Bronchology, Tokyo March 2008）。另一方面，恶性结节增生血管为卷曲状。

大量研究报道了EBUS-TBNA在诊断结节病时的有效率，其对肉芽肿的诊断率为80%-90%^[27-29]^。其中大多数病例像Nakajima等前瞻性研究所示，为1期结节病，患者同时行EBUS-TBNA和TBLB。EBUS-TBNA敏感性明显高于TBLB，在2期和3期结节病中EBUS-TBNA是否取代TBLB仍在研究中。已在进行中的随机研究将回答这一问题。

Tremblay等报道了一项对比EBUS-TBNA（22 G）和盲法TBNA（19 G）对结节性淋巴结的研究结果，其敏感性分别为96%和73%，存在统计学差异，支持EBUSTBNA^[30]^。这些作者质疑1期结节病患者行TBLB的必要性，因其增加了气胸和出血的危险性。

例5  良性淋巴结的活检

65岁，老年男性，偶然发现左下叶（图 6a）3 cm× 2 cm病变，痰细胞学显示为鳞状细胞癌。在丙型肝炎随访过程中曾行胸部X线检查。吸烟史90包年，肺功能正常，胸部CT检查显示隆突下及双侧肺门大量小淋巴结。因此行PET扫查，显示（图 5b）左下叶肺癌原发病灶高摄取，而在肺门及纵隔区域为大量中等摄取的多发对称淋巴结，PET显示淋巴结征象为良性病变，但肺叶切除前须明确其组织学诊断。于11L、7L和4R区行EBUSTBNA取样，所有淋巴结最大径小于1 cm，所有标本显示仅有充满碳末的巨噬细胞和一些淋巴细胞，未见恶性细胞。其随后行肺叶切除术，并确诊为3 cm大小的鳞癌，肺门及9区淋巴结活检良性，这些淋巴结显示碳末沉着和纤维化。

**6:**
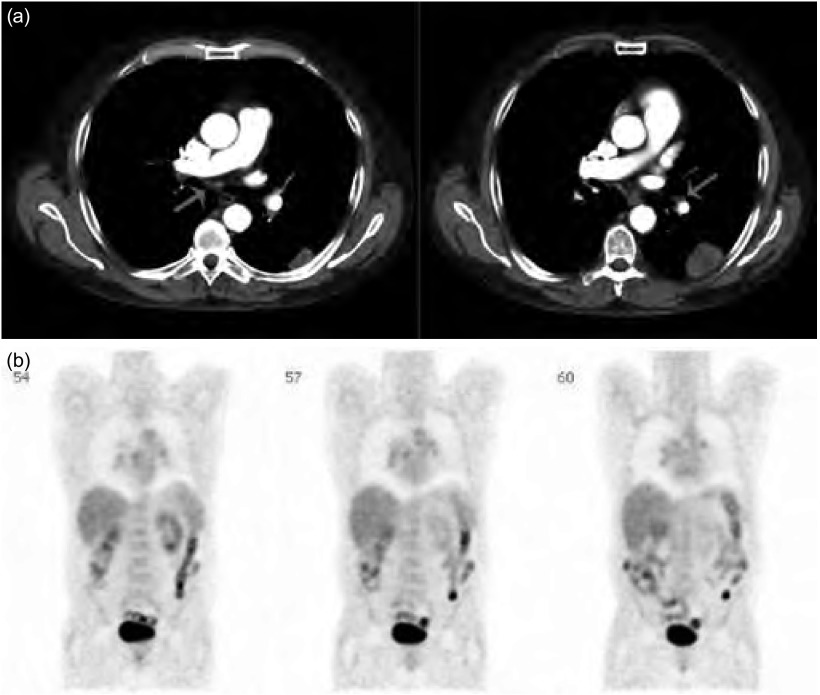
例5。（a）：胸部CT图像；（b）：PET图像显示外周肿块、多方肺门、纵膈淋巴结阳性。 Case 5. (a) CT chest. (b) Case 5 PET scan showing peripheral mass as well as multiple symmetrical hilar and mediastinal lymph node positivity.

EBUS-TBNA在此类病例中显示了巨大优势。若盲法TBNA穿刺标本为阴性时，存在未活检到指定淋巴结的可能性。但EBUS-TBNA图像可确定穿刺针的部位，大大减少了不确定性，随后，若活检组织中含有中到大量淋巴细胞，即可认定该结节为良性。恶性结节总的假阴性率为4%-8%，稍高于小淋巴结分期的病例^[7, 11]^。有时（如本例）阴性结果通过外科切除得以确认。支持良性诊断的其它因素包括大量含碳或二氧化硅物质，如果可行PET检查，看到包括肺门的双侧对称的低至中等阳性淋巴结有助于诊断，我们经常见到此类有粉尘和二氧化硅职业接触史的患者须行异常肺门或纵隔淋巴结的组织学诊断，对于这些患者，如果外科手术未确诊为良性病变，根据情况6个月-12个月内必须复查CT。

## 并发症

EBUS-TBNA是一项安全的技术，未见严重的不良反应报道，与盲法TBNA一样，主要不良反应是对支气管镜活检通道的破坏。这常发生于穿刺针前进时，其针鞘并未伸出活检通道外。正确地学习使用穿刺针是十分重要的。最近有2例类似支气管囊肿或甲状腺囊肿的EBUSTBNA后囊性结构感染的报道^[31, 32]^，如果穿刺后仍有残留液体将导致感染。此类病例最好术后给予抗炎治疗。如果液体完全吸出即不会发生感染^[33]^。当EBUS图像可确定为液性暗区，最好不要穿刺这些淋巴结肿块。另一不良反应来自于作者早期经历。40岁男性PET扫查多发淋巴结阳性，行4R及隆突下淋巴结穿刺，局部麻醉下行穿刺，患者剧烈咳嗽，在一次采用猪背法穿刺时，患者咳嗽加剧，导致TBNA针头折断，针尖残留于支气管壁，马上使用支气管活检钳将其完全取出，无持续不良反应。自此我们改良方法，避免使用猪背法，并在患者剧烈咳嗽时缩短操作过程。还有1例EBUS-TBNA后气胸报道。

## 结论

EBUS-TBNA有广泛适应征，它已经成为支气管镜技术必要的组成部分，并在肺癌分期中占据重要地位，常取代纵隔镜。所有经验丰富的支气管镜医师在20例-30例练习后可掌握这一技术。
